# Social knowledge and social reasoning abilities in a neurotypical population and in children with Down syndrome

**DOI:** 10.1371/journal.pone.0200932

**Published:** 2018-07-20

**Authors:** Koviljka Barisnikov, Fleur Lejeune

**Affiliations:** Child Clinical Neuropsychology Unit, FPSE, University of Geneva, Geneva, Switzerland; University of L'Aquila, ITALY

## Abstract

Social knowledge refers to the ability to analyze and reason about social situations in relation to social rules which are essential to the development of social skills and social behavior. The present research aimed to assess these abilities with the “Social resolution task” in a neurotypical population of 351 children (4 to 12 years) and 39 young adults, and in 20 participants (10 to 18 years) with Down syndrome. Results showed that young children aged 4 to 6 were well able to distinguish between appropriate and inappropriate social behavior but they had significantly more difficulties in judging and identifying social cues for the transgression of conventional rules than for moral ones. Between age 4 and 8, their social reasoning was mainly based on factual answers, while older children showed significantly more social awareness, making more reference to emotional and social consequences for the “victims”. The representation of a more universal applicability of social rules seemed to develop later in childhood, as of age 8. In contrast, participants with Down syndrome exhibited significantly more difficulties in judging, identifying and reasoning about transgression of social rules without social awareness. In conclusion, the results have shown that social reasoning abilities develop throughout childhood. Social awareness seems to have a long developmental course, which includes a sensibility about welfare and intersubjectivity, critical for the development of prosocial behavior. The clinical population with difficulties in social interaction and socio-emotional behavior could benefit from an early assessment and from learning social reasoning abilities to improve social skills.

## Introduction

Development of social skills depends largely on high order socio-cognitive abilities [1] including the ability to make inferences about other people’s intentions, emotions and thoughts. Social knowledge is one of the most important social competences and could be defined as the ability to analyze and reason about social situations in relation to social rules. This plays a crucial role in the understanding of how the social world is organized and regulated. The correct understanding and judgment of one’s own and others’ behavior influence the selection of the behavioral response to a situation [2,3]. Developmental, clinical and social psychology in particular have taken a long-standing interest in studying social reasoning abilities and their relation to behavior [2,4]. For example, social reasoning has been linked to specific aspects of prosocial-oriented behavior (e.g. sharing, cooperation, empathy) allowing for successful interpersonal relationships [5,6]. Moreover, difficulties in social reasoning have been associated with internalizing and externalizing behavior, peer rejection [7–9] and more specifically with aggression and social anxiety [10]. This research will focus on the development of social reasoning in relation to social rule knowledge.

The most widely accepted social domain theory suggests that social reasoning is broadly organised within moral/conventional rule distinction [11–13]. The transgression of moral rules is defined by its consequence for the right and welfare of others. Moral rules are context-free and can be judged on “behavior action” as such [13]. In contrast, the transgression of conventional rules is authority- and context-dependent and is related to a violation of conventional proscriptions, such as consensus, rules and authority [13]. Moral transgressions are generally judged by neurotypical (NT) children as more serious and less permissible than conventional transgressions [4,14]. A recent neuroimaging study revealed the existence of a core set of regions that processes social rules in general comprising judgments of conventional and moral rules and that reflects valence-based decision-making [15]. However, judgments of the two types of social rules also involved differential responsiveness of cerebral areas, supporting the theoretical distinction between moral and conventional rules. Moreover, a behavioral study revealed that a lower capacity to differentiate moral and conventional rules was associated with proactive aggressive behavior in 4- to 6-year-old NT children [16]. These studies confirm the importance of this moral/conventional distinction when considering social reasoning abilities.

The literature has reported that the ability to reason about moral and conventional rule transgressions develops from a young age into later childhood and adolescence. It has been documented that very young children in their first years of life react to the transgression of moral rules and are sensitive to someone else’s distress [17,18]. Studies have also provided evidence that 4- and 5-year-old children already understand basic moral rules related to equality, fairness and justice [13]. By age 5, children distinguish between different domains of social knowledge, which coincide with formal schooling [19]. A linear improvement in moral reasoning from 6 to 20 years was observed in NT children and adolescents [20]. The authors observed a significant group difference between childhood (6 to 8 years) and preadolescence (9 to 11 years) consistent with the rapid cerebral development during preadolescence in fronto-temporal circuits [21]. Another important group difference was found between early adolescence (12 to 14 years) and middle adolescence (15 to 17 years), indicating that moral reasoning continues to develop during adolescence. Finally, moral reasoning development slows down after the age of 18. The link between the difficulties in judging the transgression of social rules and problematic behavior has been reported in children [22], as well as in adults [3,23], resulting in a growing interest for the study of social knowledge.

Studies were particularly interested in children and adults with developmental disorders presenting difficulties in social interaction and in socio-emotional behavior especially in the population with autism spectrum disorders (ASD) [24,25–28]. In order to study these at-risk populations, some authors have developed or adapted assessment material. Barisnikov, Van der Linden and Hippolyte [29] developed the Social Resolution Task (SRT) which aimed to assess abilities to judge, identify and reason about others’ behavior in relation to conventional and moral rule knowledge. It consisted of fourteen colored pictures of everyday appropriate (for example, sharing) and inappropriate (for example, destroying) social situations. Each picture illustrated one nonverbal social scene, showing the context and the protagonist’s behavior. Similarly to the Loveland et al. [27] procedure, participants were asked three questions: to judge if the situation was appropriate or not, to identify which part of the event was right/wrong and to explain why it was right/wrong (answers were coded into three categories). The first category corresponded to an irrelevant answer showing a misunderstanding of the situation. The second category was a factual answer limited to a simple description of the picture. The third category answer was based on causality relations with social awareness. Finally, the fourth category of answer was related to a concept of social rules that is not exclusive to the situation. Several studies using the SRT reported difficulties in children and adults with different developmental disorders, in comparison with their control peers.

Indeed, Lejeune et al. [30] compared social reasoning abilities between very preterm born children aged 5 to 7 years with their full-term born peers. Results showed that preterm children showed difficulties in understanding and reasoning about inappropriate social behavior. They used more irrelevant information and exhibited less social awareness when reasoning about the transgression of social rules. Furthermore, a structural brain connectivity study [31] using the SRT in 6-year-old preterm children reported correlations between weaker connections of medial and orbitofrontal networks and lower social reasoning abilities, as well as higher externalizing behaviors (high hyperactivity symptoms) in extremely preterm (GA <28 weeks) children. These networks have been identified as important for social cognition abilities [32]. The SRT has also been used in adults with Down Syndrome (DS; mean age of 32.2 years) and the results have shown that their overall performance was like that of young NT children (mean age of 5.6 years), but the group with DS identified significantly fewer inappropriate situations than the control group. [33]. Finally, the SRT was also proven to be sensitive and a good measure for the assessment of the socio-emotional intervention effect. Indeed, after participating in a socio-emotional re-education program, a significant improvement on the social reasoning sub-score was observed in adults with intellectual disabilities of non-specific origin [34] and with DS [35]. Similarly, after social cognition training, an improvement in social reasoning abilities on the SRT was associated with higher social competence [36,37] and social information processing abilities [38] in preschool children with externalizing behavior disorders. Taken together, these studies show that the SRT is well adapted for assessing children and adults with developmental difficulties with and without intellectual disabilities. However, the results on NT children provided limited information on the developmental course of these abilities as they were presented in comparison with clinical populations including only children aged 4 to 7 years.

Therefore, it is of great importance to assess a large age range of a NT population, in order to identify critical periods of development for these different abilities assessed with the SRT. Such information could be particularly useful for understanding and interpreting the performances of child and adult clinical populations. As seen above, the understanding and the judgement of one’s and other’s behaviour could be related to social behaviour difficulties in different developmental disorders [6] and in particular among those with intellectual disabilities (ID) [27,28,33]. Individuals with DS, the most common genetic cause of ID, are characterised by prosocially oriented behaviour, offering a unique opportunity to study the social knowledge in the context of ID. The results of adults with DS were comparable to those of young NT children (mean age of 5.6 years), indicating the presence of a developmental delay in social reasoning abilities [33], but no information is available in children with DS. Consequently, studying these abilities in a younger population of children with DS would also help to further characterize the developmental trajectory of social reasoning abilities in people with DS.

The present research, composed of two studies, had two main aims: (1) to assess the developmental trajectory in social reasoning abilities by using the SRT in NT children aged 4 to 12 years and in young adults (study 1); (2) to assess the usefulness of these results in the clinical population, by comparing the performance of children with DS with those of the NT population (study 2).

## General method

### Procedure

The children were assessed individually in a quiet room at their school for about 20–30 minutes, while the young adults were assessed in our laboratory at the university. The study was conducted by using the Social Resolution Task (SRT) [29] and the French adaptation of the Peabody Picture Vocabulary Test-Revised [39].

#### Social Resolution Task (SRT)

**Fig 1:**
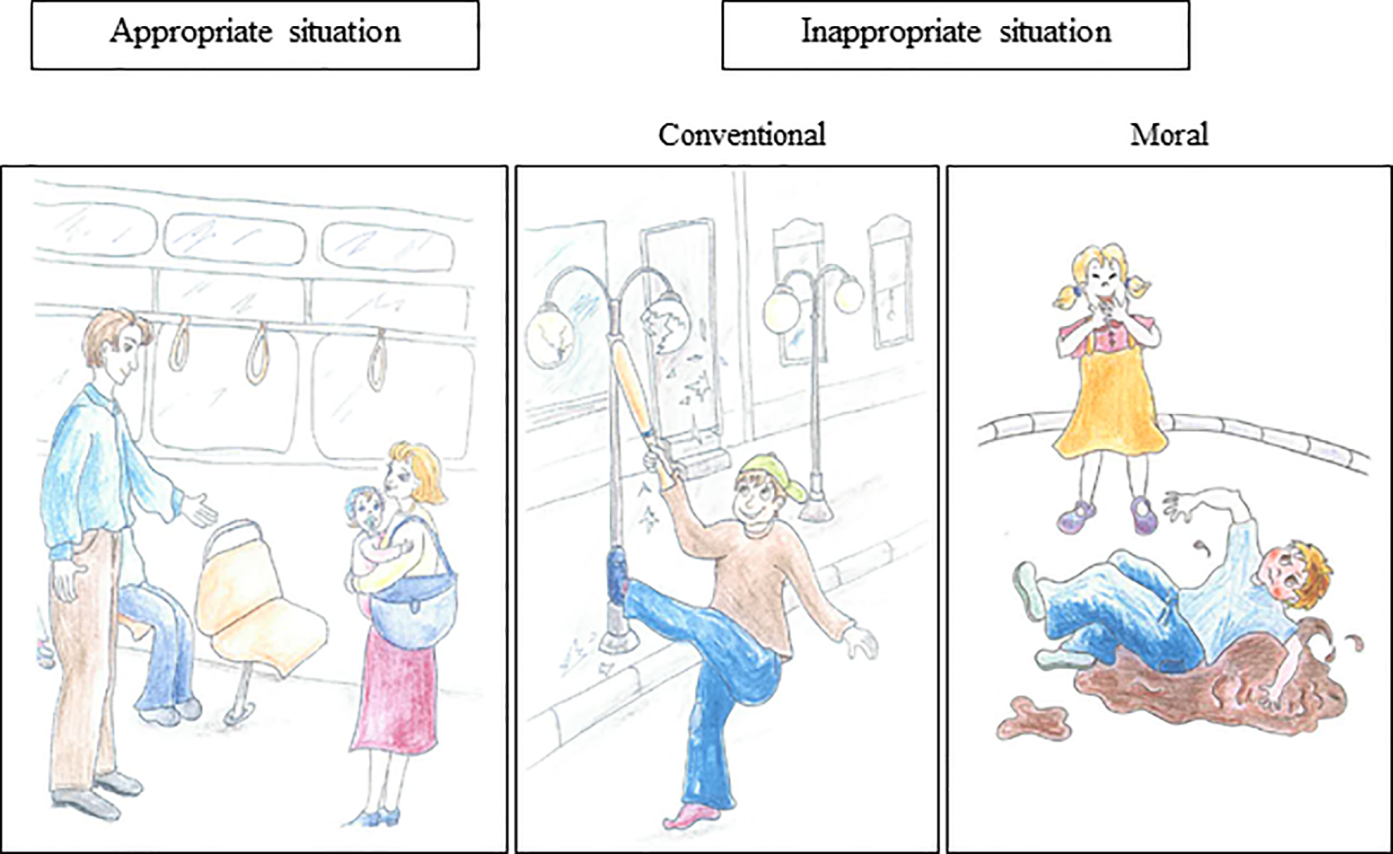
Example of pictures for the *Social Resolution Task (SRT)*.

The SRT was developed to assess social reasoning skills by judging, identifying and reasoning about conventional and moral rule knowledge [29]. It was composed of fourteen colored drawings illustrating everyday social situations: five of them illustrated appropriate situations (e.g. helping, cooperating, or sharing) and nine of them, inappropriate situations (e.g. bothering, not sharing, or destroying). Five of the nine inappropriate situations were related to a violation of moral rules and the other four, of conventional rules (Fig 1).

The scoring system was initially adapted from the Loveland et al.’s study [27]. As the main goal of the SRT is to assess social rule knowledge, the scoring system was adapted to promote the abilities of judging the appropriateness of the social scene and to distinguish the different levels of social reasoning. Accordingly, the judgement and social reasoning sub-scores have more impact on the global SRT score. In contrast, the identification sub-score had less impact on the global SRT score because it only provided information on the element on which the participant relied to judge the appropriateness/inappropriateness of the scene. Moreover, the scoring system was the same as in previous studies that used SRT [30,31,33,34,36–38].

For each situation, 3 questions were asked to the child: (1) “Is this situation correct or incorrect?”, (2) “Show me what is correct/incorrect”, (3) “Why is it correct or incorrect?”. For the first question (Q1) assessing the ability to distinguish between appropriate /inappropriate situation, 2 points were given for each situation if the participant correctly judged the appropriateness/inappropriateness of the situation, if not, a 0 was given (judgement sub-score). For the second question (Q2) providing information about the visual cue used for judging the social situation,1 point was given if the participant identified the relevant element in the picture (identification sub-score). For the third question (Q3), only the 9 inappropriate situations were taken into account and classified into 4 response categories with an increase of the score corresponding to the participant’s level of social reasoning (social reasoning sub-score): (1) Irrelevant answer, 0 points for an incorrect or inappropriate answer; (2) Factual answer, 2 points for a description of the scene without social awareness; (3) Intersubjective answer, 5 points for an answer based on causality relations with social awareness; (4) Conceptual answer, 7 points for an answer based on conceptual knowledge of conventional or moral rules. The different levels of answers are described in Table 1. A global SRT score and 3 sub-scores, 1 for each question, were calculated in percentages. Moreover, separate scores were calculated for the appropriate and inappropriate situations. Participants’ answers were coded separately by two independent experimenters.

**Table 1:** Brief description of the different levels of answers for the third question (social reasoning score).

Category (points)	Brief description	Example: a boy falls in the mud in front of a girl and she laughs
Irrelevant (0)	Incorrect or inappropriate answer	“She pushed him in the mud, it's mean”
Factual (2)	Description of the scene without social awareness	“the boy falls in the mud and the girl laughs”
Intersubjective (5)	Answer based on causality relations with social awareness	“The boy will be embarrassed because she laughs”
Conceptual (7)	Answer based on conceptual knowledge of conventional or moral rules	“We do not make fun of someone in a difficult situation”

#### Peabody Picture Vocabulary Test-Revised

Receptive vocabulary knowledge was assessed using the standardized EVIP vocabulary scale, a French adaptation of the Peabody Picture Vocabulary Test [39]. In this test, participants hear a word spoken by the experimenter, and they have to select out of four pictures the one that matches the spoken word. Test administration is stopped after six erroneous responses over eight consecutive trials. As a dependent variable, the raw vocabulary score (number of the item at which the test was stopped minus errors) was retained for analysis. This task has a high test-retest reliability [r = .80; 39].

## Study 1: Developmental trajectory in social reasoning abilities in NT children age 4 to 12 and in young adults

### Participants

The participants were children aged between 4 to 12 years and young adults. There were 390 in total and they were divided into 10 age groups of 39 each: Children with developmental and learning difficulties were excluded from the study. They were recruited through local public schools in Geneva after the parents received an information letter and a consent form about the study. An overall description of the population is presented in Table 2. The Ethical Committee of the University of Geneva approved the study and the Cantonal Authorities for Primary Education as well as the school administration authorities delivered the authorization. All participants were volunteers and could leave the study at any time.

**Table 2:** Participants’ characteristics.

Age group	N	Age; M(SD)	Gender ; % girls	EVIP-R; M(SD)
4 years	39	4.58 (0.2)	46.2	55.9 (17)
5 years	39	5.44 (0.35)	56.4	66.8 (15.6)
6 years	39	6.51 (0.3)	41	85.7 (18.4)
7 years	39	7.44 (0.26)	46.2	95.5 (18.7)
8 years	39	8.36 (0.32)	48.7	103.6 (15.9)
9 years	39	9.48 (0.3)	53.8	115.5 (12.1)
10 years	39	10.47 (0.34)	43.6	115.4 (15.5)
11 years	39	11.5 (0.33)	56.4	127.5 (16.3)
12 years	39	12.41 (0.24)	43.6	135.3 (12.2)
Adults	39	24.29 (3.21)	76.9	166.7 (5.8)

### Statistical analysis

All statistical analyses were conducted using SPSS 22.0 (IBM SPSS Statistics, IBM Corporation). For the global SRT score and the appropriate situations (judgement and identification sub-scores), analyses of variance (ANOVA) were performed with the age group as a between-subjects factor (4 years to adults). For the inappropriate situations (judgement, identification and social reasoning sub-scores), ANOVAs were performed with the age group (4 years to adults) as a between-subjects factor and the type of social rules (moral vs. conventional) as a within-subject factor. In order to identify critical periods of development between ages, contrasts were then used to compare two consecutive age groups. The significant threshold was 0.05.

### Results

#### Global SRT score

Results are presented in Table 3. The results of the ANOVA revealed a significant effect of the age group, *F*(9,380) = 66.72, *p* < .001, *η*^2^_p_ = .612. Children had significantly better global SRT scores as they grew up. Regarding the important number of comparisons (n = 9), a Bonferroni alpha-level correction was adopted (α = 0.05/9 = 0.0056) for the contrasts. Results revealed three critical periods of improvement: between 4 and 5 years (*p* = .003), between 10 and 11 years (*p* = .004) and between 12 years and the adult group (*p* < .001). Analyses were then performed on the 3 SRT sub-scores.

**Table 3:** Mean scores (percentage) and standard deviations for the SRT scores according to age group and social rule.

Age group	Global SRT	Q1 : Judgement			Q2 : Identification			Q3 : Social Reasoning
		Appropriate	Inappropriate		Appropriate	Inappropriate		
			Moral	Conventional		Moral	Conventional	
4 years	50.9 (6.3)	84.6 (22.7)	86.2 (17.9)	73.7 (22.9)	74.4 (25.5)	76.9 (19.8)	67.3 (23.8)	27.5 (8.8)
5 years	56.2 (8.4)	89.2 (23.3)	93.3 (12.4)	87.2 (16.1)	88.2 (22.3)	88.2 (17.6)	83.3 (19.3)	32 (11.4)
6 years	60.8 (9.3)	97.4 (6.8)	93.8 (11.4)	96.2 (9.1)	91.3 (18.2)	89.7 (13.7)	93.6 (11.1)	38 (16.2)
7 years	62.5 (8.3)	97.4 (6.8)	96.9 (8.6)	95.5 (9.7)	97.4 (6.8)	95.4 (8.5)	92.9 (12.8)	39.4 (13.1)
8 years	65.2 (6.7)	98.5 (7.1)	95.9 (10.4)	96.8 (8.5)	97.9 (7.7)	95.4 (10.7)	96.8 (8.5)	43.3 (11.5)
9 years	68.7 (7.8)	100 (0)	98.5 (5.4)	96.8 (8.5)	99.5 (3.2)	97.4 (6.8)	92.9 (11.4)	48.6 (12.9)
10 years	67.9 (8.3)	100 (0)	99.5 (3.2)	98.7 (5.6)	99.5 (3.2)	99.5 (3.2)	98.1 (6.7)	47.1 (13.6)
11 years	73.1 (7.7)	99.5 (3.2)	99.5 (3.2)	97.4 (7.7)	99.5 (3.2)	97.4 (6.8)	95.5 (9.7)	56 (12.4)
12 years	75.5 (8.5)	98.5 (5.4)	100 (0)	96.8 (8.5)	98.5 (5.4)	98.5 (5.4)	96.8 (8.5)	60.2 (14)
Adults	87.5 (7.7)	98.5 (5.4)	99 (4.5)	96.2 (9.1)	97.9 (6.1)	98.5 (5.4)	94.9 (11.7)	80.6 (12)

#### Q1: Judgement

For appropriate situations, results showed a significant age group effect, *F*(9,380) = 8.21, *p* < .001, *η*^2^_p_ = .163 (see Table 3). A Bonferroni alpha-level correction was adopted for the contrasts (α = 0.05/9 = 0.0056). Results revealed one critical period of development between 5 and 6 years (*p* = .001), indicating an improvement of performance until reaching a ceiling effect at age 6.

For inappropriate situations, results revealed significant effects of age group, *F*(9,380) = 20.81, *p* < .001, *η*^2^_p_ = .330, of type of social rules, *F*(1,380) = 15.63, *p* < .001, *η*^2^_p_ = .040, and a significant age group × type of social rules interaction, *F*(9,380) = 3.54, p < .001, *η*^2^_p_ = .077. A Bonferroni alpha-level correction was adopted for the contrasts (α = 0.05/28 = 0.0018). For moral rules, results revealed one critical period of development between 4 and 5 years (*p* < .001). For conventional rules, two critical period of development were observed: between 4 and 5 years (*p* < .001) and between 5 and 6 years (*p* < .001). Moreover, children had significantly more difficulties in judging the inappropriateness of a situation for conventional rules than for moral ones at 4 years (*p* < .001). A ceiling effect was observed at 7 years.

#### Q2: Identification

A significant age group effect was observed, *F*(9,380) = 14.86, *p* < .001, *η*^2^_p_ = .260 for appropriate situations (see Table 3). A Bonferroni alpha-level correction was adopted for the contrasts (α = 0.05/9 = 0.0056). Results indicated a significant increase between 4 and 5 years (*p* < .001), followed by a ceiling effect by age 7.

For inappropriate situations, significant effects of age group, *F*(9,380) = 26.88, *p* < .001, *η*^2^_p_ = .389, of type of social rule, *F*(1,380) = 10.59, *p* = .001, *η*^2^_p_ = .027, and a significant age group × type of social rule interaction, *F*(9,380) = 2.29, *p* = .016, *η*^2^_p_ = .051, were found. A Bonferroni alpha-level correction was adopted for the contrasts (α = 0.05/28 = 0.0018). For moral rules, one critical period of development between 4 and 5 years was observed (*p* < .001). For conventional rules, results indicated two critical period of development: between 4 and 5 years (*p* < .001) and between 5 and 6 years (*p* < .001). Moreover, 4-year-old children had significantly more difficulties in judging the inappropriateness of a situation for conventional rules than for moral ones (*p* < .001). A ceiling effect was observed at 8 years.

#### Q3: Social reasoning

Results revealed a significant age group effect, *F*(9,380) = 58.01, *p* < .001, *η*^2^_p_ = .579 (see Table 3). Children had significantly better social reasoning scores as they grew up. A Bonferroni alpha-level correction was adopted for the contrasts (α = 0.05/9 = 0.0056). Results showed two critical periods of improvement: between 10 and 11 years (*p* = .003) and between 12 years and the adult group (*p* < .001). No other significant effect was observed.

**Fig 2:**
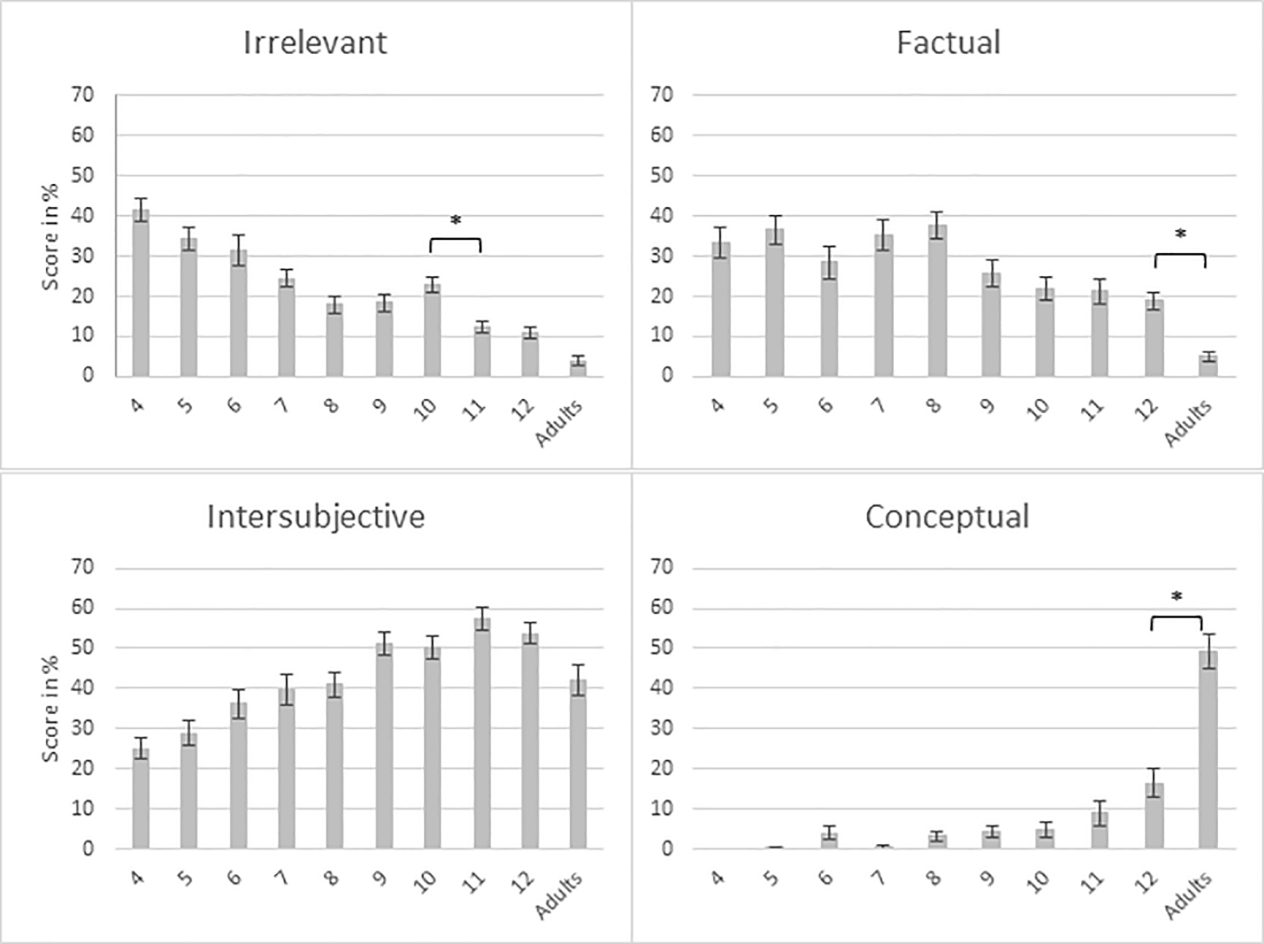
Mean social reasoning scores (percentage) and standard errors, according to age group (age 4 to adults) and to the category of responses (irrelevant, factual, intersubjective, conceptual).

To further investigate the results of social reasoning, the percentage of answers in each category was analyzed. ANOVAs were thus performed with age group as a between-subjects factor for each category. The results are presented in Fig 2. A significant age group effect was observed for each category. A Bonferroni alpha-level correction was adopted for the contrasts (α = 0.05/9 = 0.0056). (1) The youngest children gave irrelevant answers more often than the older ones and the adults, *F*(9,380) = 25.32, *p* < .001, *η*^2^_p_ = .375. Contrasts showed one critical period of decrease between 10 and 11 years (*p* = .001). (2) The children often gave factual answers between 4 and 8 years, then from the age of 9 they gave them less and less often, *F*(9,380) = 10.10, *p* < .001, *η*^2^_p_ = .193. Contrasts revealed one critical period of decrease between 12 years and the adult group (*p* = .002). (3) They gave significantly more intersubjective answers as they grew up, then there was a decline at age 12 and in the adult group, *F*(9,380) = 11.40, *p* < .001, *η*^2^_p_ = .213. (4) Moreover, there was a slight increase of conceptual answers during childhood, *F*(9,380) = 44.42, *p* < .001, *η*^2^_p_ = .513, followed by a significant improvement between age 12 and adulthood (*p* < .001).

## Study 2: Comparison of social reasoning abilities between children with DS and NT children

### Participants

Twenty participants with DS (10 girls and 10 boys) between 10 and 18 years of age (M = 13.7 years, SD = 2.14) were included in the study. Two participants failed to complete the SRT and were excluded from the study. They were recruited through special education schools after the parents received an information letter and a consent form about the study. They were matched individually for gender and raw score on the vocabulary task (EVIP-R) with a control group of 20 NT children (10 girls and 10 boys) selected from Experiment 1. The mean age of the control group was 5.5 years (SD = 1.26, range from 4.1 to 8.7 years). The mean raw scores on the vocabulary task were 51.9 (SD = 21) for the children with DS and 52.6 (SD = 21) for the NT children.

The Ethical Committee of the University of Geneva approved the study and the Cantonal Authorities for Primary Education as well as the school administration authorities delivered the authorization. All participants were volunteers and could leave the study at any time.

### Statistical analysis

All statistical analyses were conducted using SPSS 22.0 (IBM SPSS Statistics, IBM Corporation). For the global SRT score, an ANOVA was performed with the group as a between-subjects factor (NT vs. DS). For the appropriate situations, an ANOVA was conducted with the group (NT vs. DS) as a between-subjects factor, and the type of questions (Q1: judgement vs. Q2: identification) as a within-subject factor. For the inappropriate situations, an ANOVA was performed with the group (NT vs. DS) as a between-subjects factor, and the type of social rules (moral vs. conventional) and the type of questions (Q1: judgement vs. Q2: identification) as within-subject factors. Finally, for the social reasoning score, an ANOVA was conducted with the group (NT vs. DS) as a between-subjects factor, and the type of social rules (moral vs. conventional) as a within-subject factor. The significant threshold was 0.05.

### Results

#### Global SRT score

Results are presented in Table 4. The ANOVA revealed a significant effect of the group factor, *F*(1,39) = 17.7, *p* < .001, *η*^2^_p_ = .317. NT children had significantly better global SRT scores than children with DS.

**Table 4:** Mean scores (percentage) and standard deviations for the SRT scores according to the population.

Group	Global SRT	Q1: Judgement		Q2: Identification		Q3: Social Reasoning
		Appropriate	Inappropriate	Appropriate	Inappropriate	
NT group	54.3 (7.4)	88 (15.1)	86.1 (13.4)	86 (16)	83.9 (13.7)	30.3 (14)
DS group	41.8 (11)	70 (35.2)	73.3 (25.3)	59 (31.4)	60.6 (24)	17.8 (11.1)

#### Q1: Judgement and Q2: Identification

For appropriate situations, results revealed significant effects of group, *F*(1,38) = 7.8, *p* = .008, *η*^*2*^_*p*_ = .170, of type of questions, *F*(1,38) = 14.9, *p* < .001, *η*^*2*^_*p*_ = .282, and a significant group × type of questions interaction, *F*(1,38) = 7.2, *p* = .011, *η*^*2*^_*p*_ = .159 (see Table 4). The judgement and identification scores were higher for the NT group than for the group with DS. Moreover, the group with DS had significantly better judgement scores than identification ones (*p* > .001), while no significant effect was observed in the NT group.

For inappropriate situations, results revealed significant effects of group, *F*(1,38) = 8.9, *p* = .005, *η*^*2*^_*p*_ = .190, of type of questions, *F*(1,38) = 21.01, *p* < .001, *η*^*2*^_*p*_ = .356, and a significant group × type of questions interaction, *F*(1,38) = 9.8, *p* = .003, *η*^*2*^_*p*_ = .204 (see Table 4).The NT group had a significantly higher judgement and identification scores than the group with DS. Contrary to the NT group, the group with DS had lower identification scores than judgement scores (*p* < .001). No other significant effect was observed.

#### Q3: Social reasoning

**Fig 3:**
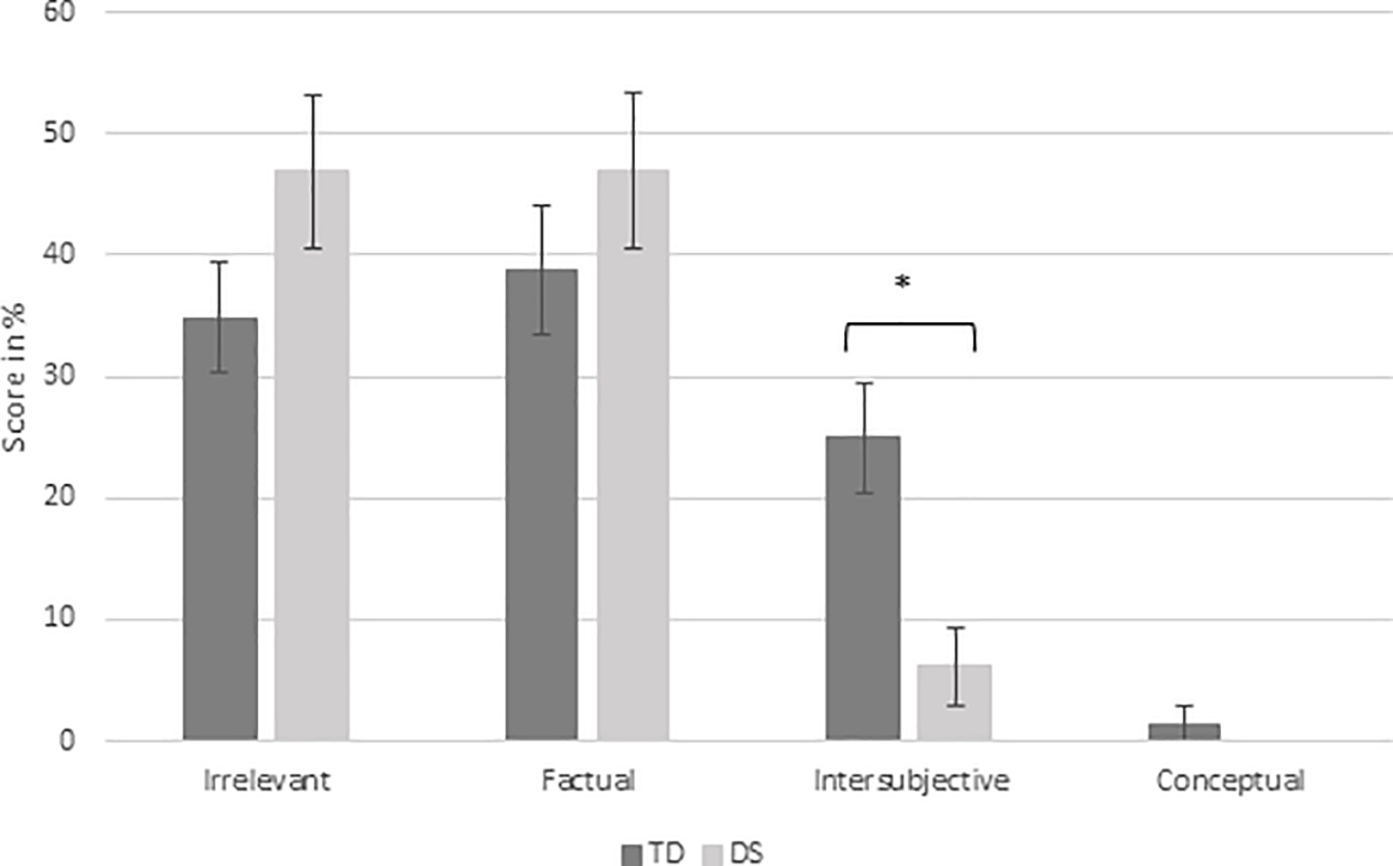
Mean social reasoning scores (percentage) and standard errors, according to the group and to the category of responses.

Results revealed a significant group effect, *F*(1,38) = 8.9, *p* = .005, *η*^2^_p_ = .191 (see Table 3). NT children had significantly better social reasoning scores than children with DS. In order to further investigate the results of social reasoning, the percentage of answers in each category was analyzed. The results are presented in Fig 3. Results showed that the NT children gave intersubjective answers significantly more often than the children with DS, *F*(1,38) = 11.4, p = .002, *η*^2^_p_ = .231. No other significant effect was observed.

## Discussion

The present study aimed first to assess the developmental trajectory in social reasoning abilities by using the SRT in NT children aged 4 to 12 years and in young adults (study 1). The second aim was to assess the usefulness of the developmental results in the clinical population, by comparing the performance of children with DS with those of the NT population (study 2). The results on the NT population will be presented first, following by the results of the children with DS.

Results of study 1 revealed the existence of different critical periods for the development of the abilities to judge, identify and reason about social behavior in the NT population. Regarding the global SRT score, the results showed three critical periods of improvement: between 4 and 5 years, between 10 and 11 years and between age 12 and the adult group.

As they grow up, children improve their ability to correctly judge the appropriateness/inappropriateness of social situations (e.g. sharing; helping) and identify the pertinent cues on the picture illustrating social situations, especially between 4 and 6 years. However, 4-year-old children exhibit significantly more difficulties in judging the inappropriateness of a situation for conventional rules than for moral ones. Results also revealed one critical period of development between 4 and 5 years for moral rules, while two consecutive critical period of development were observed from 4 to 6 years for conventional rules. This difference between the two types of social rules disappears at age 6. This last result is in line with previous findings showing that the judgement of moral rule violation develops early and elicits a more natural reaction [13,14]. Whereas the judgement of the transgression of conventional rules demands a more complex and flexible comprehension of social situations [4,40], as they are context and authority dependent [11–13]. The difference in time course between these two social rules may be related to the development of higher-order cognitive processes such as attention and executive functions, which are probably more instrumental in analyzing situations involving the transgression of conventional rules. Indeed, the most impressive change in executive function abilities occurs between 3 and 5 years [41]. This hypothesis is consistent with a recent study which revealed a developmental delay in the acquisition of conventional rules in 5- to 7-year-old preterm children [30], who display generally more executive difficulties [42,43] compared to their full-term peers.

For the appropriate situations, ceiling effects are observed at 6 years for the judgment score and at 7 years for the identification score. Six-year-old NT children correctly judge the appropriate situations as well as the adults, but it is only at age 7 that they are able to identify the relevant cues as accurately as adults. For the inappropriate situations, the same developmental improvement is observed for the judgement and the identification, except that the ceiling effect is reached later, at the age of 8 years. This result indicates that it is more demanding for young children to correctly judge an inappropriate situation than an appropriate one. The age of 6 years corresponds to entry into primary school where social interaction and learning dramatically increase, allowing children to experience various social situations and to attain a certain degree of expertise in the judgement and identification of social cues. Moreover, the ceiling effect observed at 8 years for the identification score could be linked to the development of the theory of mind (ToM), which is defined as the ability to make inferences about other people's intentions, thoughts and emotions [44]. Indeed, for some first order ToM tasks (e.g. false-belief) children also reached a ceiling effect at age 8 [45,46]. At this age, children seem to become more expert in identifying social cues and in particular, in processing emotional expression and the context of various social situations.

The social reasoning supporting their answers regarding transgression of social rules continues to develop well beyond age 8. Indeed, a continuous increase of the social reasoning score is observed from years 4 to adulthood. The analysis of each category of answers allows for a better understanding of this improvement as children grow. Firstly, results reveal that irrelevant answers are very common between 4 and 6 years of age and then gradually decrease until adulthood, with one critical period of decrease between 10 and 11 years. It seems that younger children have more difficulties in identifying relevant cues, which could mislead their judgement and understanding of social situations. Secondly, between 4 and 8 years, children often give factual answers describing the situation without social awareness. Then from the age of 9 years, factual answers decrease continuously with an increase of answers based on social awareness between 4 and 12 years. One critical period between age 12 and the adult group was observed, and was characterized by an increase of conceptual answers. As they grow up, children use more and more reference to emotional and social consequences for the “victims” when explaining why they judged a particular behavior as inappropriate. This category of answers is based on reasoning which includes causality between protagonists’ actions and possible emotion and/or social damage, as well as ability to take others’ perspectives, reflecting sensibility for welfare and intersubjectivity [28].

The evolution of intersubjective reasoning observed from 9 years and reflecting social awareness, could be linked to the development of the ToM. Indeed, for some first order ToM tasks (e.g. false-belief) children reached a ceiling effect at 8 years [45,46], while the ability to resolve more complex (higher order) ToM tasks (e.g. the director task) continues to develop through adolescence and adulthood [47]. It is thus possible that the development of the ToM supports the expertise that children showed between 6 and 8 years in their ability to correctly judge the transgression of social rules and development of intersubjective reasoning. A recent neuroimaging study has compared cognitive and affective ToM abilities between adolescents and adults [48] and revealed that differential neural response between the adult and the adolescent groups indicates developmental changes in affective ToM processing. Adolescents activated a cerebral area involved in affective information processing (understanding emotions) moreso than did adults. It is thus likely that adolescents have more intersubjective answers because they are related to a more emotional analysis of the social situation than a conceptual one.

In line with these findings, an important decline of intersubjective answers in favour of conceptual ones is observed between children age 12 years and adults. This last category of answers is almost non-existent in children under age 8, then a slight increase is observed until 12 years. Thus, the representation (understanding) of a more universal applicability of social rules, beyond the social scenes illustrated in the SRT, seems to develop later in childhood.

Considering all of the above results together, the SRT seems to be a good instrument for assessing social reasoning during childhood, accessible to children as young as age 4. The ability to distinguish between appropriate and inappropriate social behavior (judgement score) and identify social cues (identification score) provides information about understanding social situations. As for the social reasoning score, it allows one to analyse social rule knowledge throughout four distinct categories of children’s answers regarding transgression of moral/conventional rules. However, the SRT requires complex cognitive abilities. Consequently, to better understand age-related changes in social reasoning abilities, future studies are needed to examine their relations to the development of EF (e.g. inhibition) and other socio-cognitive abilities (e.g. ToM, emotion recognition). As seen above, although significant improvement of conceptual reasoning is observed at 12 years, it would be interesting to assess children between age 13 and 18 in order to stress the full developmental trajectory of social reasoning abilities. To the best of our knowledge, this is the first study to give a detailed schedule of social reasoning development by studying it year by year making it possible to identify key periods that could be relevant for learning social skills. Considering the importance of social rule knowledge for adapted social functioning, the clinical population with behaviour problems could benefit from these results.

Indeed, the comparison between the performances of children with DS and the NT population provided several important findings. Results revealed that NT children had significantly better global SRT scores, as well as judgement, identification and social reasoning sub-scores than children with DS who were matched individually for gender and raw score on the receptive vocabulary task. A previous study using the SRT did not show any difference for the global SRT scores or for the judgment and identification sub-scores for appropriate situations between adults with DS and a similar NT control group [33]. However, similarly to our children with DS’s results, the judgment and identification sub-scores for inappropriate situations were significantly lower in adults with DS than in the NT control group. These results reveal that judging both types of situations is particularly challenging for children with DS. Moreover, while the ability to judge the transgression of conventional rules develops later than for moral ones in NT children, no effect of category of rules (moral and conventional) on DS performances is observed, suggesting that they follow a different developmental pattern.

Another interesting finding is that, contrary to the NT population, children with DS showed significantly lower identification scores than judgements scores regardless of the type of rules (moral and conventional). It is possible that difficulties in attention and executive functioning in children with DS have a negative influence on detection of social cues with a lack of flexibility for processing these different social situations [49]. The selective attention competences were significant predictors for judgement and identification scores in adults with DS [33]. However, the identification ability requires higher selective attention skills as participants have to focus their attention on the relevant cues of the social scenes. Interestingly, Lejeune et al. [30] reported that preterm children aged between 5 and 7 years had significantly lower identification scores for inappropriate situations on the SRT compared to their full-term peers. Although, these results were not related to their lower selective attention scores, the authors suggested that such difficulties were specific to the detection of social cues, rather than to the general attention deficit reported in preterm children.

Children with DS also had significantly lower social reasoning scores than NT children, mainly providing irrelevant (47%) and factual (47%) answers. Concerning factual answers, children with DS’s justifications are mainly based on general principles forbidding the depicted behavior (e.g. item “aggression”: “she is pulling her hair, you don’t do that, it is not good/not allowed”). The analysis of each category of answers showed that NT children gave intersubjective answers significantly more often (25%) than did the children with DS (6%), reflecting their better awareness of others’ rights, feelings and welfare [50]. As children with DS were matched on vocabulary measure, their lower performances could not be explained by such abilities. Thus the difficulties of children with DS could be related to their low socio-cognitive abilities (e.g. ToM, face emotion recognition). The SRT presents social scenes showing protagonists’ behavior with congruent facial emotion expressions. However, it did not help children with DS to provide intersubjective answers in contrast to NT children. Few studies have assessed these abilities in children with DS and they reported early and persisting difficulties in using emotional cues for social referencing [51,52], emotion recognition [53], and ToM tasks [54] which could explain their difficulties in social understanding [54]. Furthermore, at a neuronal level (both structural and functional), people with DS present anomalies in cerebral regions underpinning development of executive, socio-cognitive and emotion abilities [55,56]. Nevertheless, the significant difference of intersubjective answers between NT children and children with DS that we observed in Study 2, was not observed between adults with DS and NT children [33], revealing that children with DS have more difficulties in social reasoning abilities than adults with DS. This divergence of results between DS adults and DS children suggests the presence of a developmental delay that could be eliminated as they get older.

The difficulties of children with DS in giving answers reflecting social awareness is quite surprising as they are described as sociable and empathic [57]. However, their prosocial approach seems not to be supported by adapted social behavior; they experience difficulties in peer interaction, as well as establishing and maintaining stable relationships [58]. According to teachers’ reports [59], children with DS were rated as being less prosocial and more asocial than their NT peers, needing considerable assistance in getting play started, remaining involved, understanding social rules and knowing how to play with others. It seems that children with DS need specific support and explicit learning of social skills. They also present a higher level of behavior problems that are disruptive to peer interaction. Socio-emotional disorders and social functioning difficulties were largely reported in children with DS [60]. The above-mentioned difficulties in children with DS could explain their problems in learning social skills.

The literature has also reported difficulties in social reasoning abilities in other neurodevelopmental problems [61], premature birth [30], problematic behavior [22], ASD [27,28] or intellectual disability [53]. The difficulties these children have identifying relevant social cues could explain their difficulties in learning social skills. Consequently, the ability to consider the emotional and social consequences of transgression of social rules could be crucial to the development of social relations and adapted functioning. In this context, early intervention should be proposed. In regards to our results, some specific intervention strategies can be recommended, such as giving relevant cues to support detection of social information, explaining social rules that guide social interaction, and/or providing a coherent explanation of social situations that involve taking into account others’ perspectives and feelings.

In conclusion, the SRT is a well-adapted tool for assessing very young children and those with Down syndrome. The results provided a detailed developmental schedule of social understanding and social reasoning abilities allowing one to identify key periods that could be relevant for learning social skills. Children at risk for difficulties in social interaction and socio-emotional behavior, such as those with Down syndrome, could benefit from these results for early detection and intervention.
